# Picky Eating in Children

**DOI:** 10.3389/fped.2015.00041

**Published:** 2015-05-06

**Authors:** Jason Lam

**Affiliations:** ^1^Internal Medicine and Pediatrics Department, Western Michigan University Homer Stryker M.D. School of Medicine, Kalamazoo, MI, USA

**Keywords:** picky eating, children, vegetables, fruits, neophobia, food acceptance

Although there is no consistent definition of picky eating, the term is generally used to characterize children who eat a limited amount of food, have strong food preferences, have restricted intake (particularly of vegetables), and who are unwilling to try new foods (1). Picky eating is a relatively common behavioral problem. Recent studies have found that picky eating is not associated with having an eating disorder (2) and does not have a significant effect on growth (1).

## Prevalence

The prevalence of picky eating is difficult to account for, since there is no universally accepted definition. In a recent study involving 4,018 participants conducted in the Netherlands, the prevalence of picky eating was 26.5% at 18 months of age, 27.6% at 3 years of age, and declined to 13.2% at 6 years of age (3). The data from the study suggest that picky eating is usually a temporary behavior and is a part of normal development in preschool children.

## Etiology

Environmental factors play a role in taste and eating preferences. Flavors from aromatic compounds derived from maternal food consumption are transmitted into the amniotic fluid and breast milk; these flavors have strong influences in taste preferences and food acceptance later in life (4–6). An experimental study demonstrated that infants of mothers who drank carrot juice during the last trimester of pregnancy enjoyed carrot-flavored cereals more than infants whose mothers did not drink carrot juice or eat carrots (6). Human milk is composed of flavors that are a reflection of food consumed by breast-feeding mothers (7, 8). Varied diet in breast-feeding mothers produces more flavor exposure and experiences in children, which may help explain why breastfed infants are less picky (9) and more willing to try new foods (10). This notion was also supported in a recent study, in which 127 children who were exclusively breastfed for 6 months were observed to have lower odds of developing a preference for food to be prepared in a certain way by 78%, food rejection by 81%, and avoidance of new food (neophobia) by 75% (11). Genetics also play a role in picky eating. Early preferences for sweet taste have been observed in newborns (12). On the other hand, bitter taste is innately disliked, possibly due a protective mechanism since most bitter compounds are toxic (13). Hence, neophobia may be an evolutionary protective mechanism, serving to protect children from ingesting potentially toxic substances. These innate food preferences may become barriers for acceptance of certain food. A study involving 5,390 pairs of twins from 8 to 11 years of age suggested that neophobia is a highly heritable trait, meaning a child’s reluctance to try new food may be partly due to genetics and not parental practices (14).

## Promoting Food Acceptance

There are many ways to promote food acceptance among children. Mothers (particularly those who do not plan to breast-feed) are recommended to eat a variety of food during their pregnancy and then expose their child to variety of food at an early age (15). Children may exhibit normal exploratory behaviors with new foods such as touching, smelling, playing, putting foods in their mouth, and then spitting them out before they are willing to taste and swallow various foods (16). Repeated taste exposure and modeling of behaviors in non-coercive fashion has shown to increase food acceptance (10, 17, 18). Conversely, pressuring children to eat can cause them to dislike those foods (19). Researchers in a study of 3,022 infants found that many caregivers were not aware that their infants and toddlers needed as many as 8–15 exposures to a particular food before they gained acceptance of that food (20). Increased acceptance and consumption of poorly liked food by children such as nutrient-rich fruits and vegetables (21) can be achieved by offering children very small tastes of new and previously disliked fruits and vegetables (22–24). Food is also more readily accepted in young children when others around them are eating the same type of food (25, 26). Such modeling positively highlights the enjoyment of such foods. Praising children for trying new food and giving them small token rewards, such as stickers (but not treats) also increases acceptance. Some authors have argued that providing rewards for performing a task diminishes intrinsic motivation (27). However, this only applies to interesting tasks. Most children who are labeled as “picky eaters” have little interest in eating fruits and vegetables. Hence, “there is little or no intrinsic motivation to undermine” (27).

## Differential Assessment

Other causes that may appear as “picky eating” should also be taken into consideration. Lactose intolerance or food allergies can present with failure to thrive, oral pruritus, abdominal pain, vomiting, diarrhea, and refusal of specific types of food (28). Gastroesophageal reflux disease can present with frequent complaints of heartburn, vomiting, and refusal of foods given the negative association. Children with oral hypersensitivity may also develop adverse reactions to feeding due to the abnormally strong and unpleasant sensation(s) with different types of food.

## Conclusion

Picky eating is a relatively common behavioral problem that most children will eventually outgrow. Its roots stem from both environmental and genetic influences. It may serve as a protective mechanism to avoid exposure to potentially toxic substances. Increasing exposure to a variety of flavors during pregnancy and infancy (either directly or indirectly via breast milk) may reduce the incidence of picky eating. Repeated exposure to new food in a non-coercive manner and in an environment that is both fun and rewarding may help overcome picky eating. Patience, time, and repetition may be the keys to success. While more research is needed on this topic, it is reasonable to recommend that healthcare providers offer reassurance and education to parents who are concerned about picky eating in their child after they have assessed for any treatable causes. Education is particularly important in parents who plan to have additional children in the futures.

## Conflict of Interest Statement

The author declares that the research was conducted in the absence of any commercial or financial relationships that could be construed as a potential conflict of interest.
